# Peer Review of “Patterns of Physical Activity Among University Students and Their Perceptions About the Curricular Content Concerned With Health: Cross-sectional Study”

**DOI:** 10.2196/37322

**Published:** 2022-04-29

**Authors:** David Salman

**Affiliations:** 1 Imperial College London London United Kingdom

**Keywords:** physical activity, university students, university, exercise, students, inactive, curricula, healthy lifestyle, higher education

*This is a peer-review report submitted for the paper “Patterns of Physical Activity Among University Students and Their Perceptions About the Curricular Content Concerned With Health: Cross-sectional Study.”*

## Round 1 Review

### General Comments

It is very positive to see analysis of physical activity in different populations and different age groups, and this paper [1] is a very welcome study in terms of physical activity in India and in relation to students. This is an important area as, when trying to engender habits and physical activity across the lifespan, it is in the younger age groups where sustained impact can be made. However, I feel that this paper addresses the issue quite superficially and would benefit from more in-depth analysis.

### Specific Comments

#### Major Comments

1. Throughout the paper, there is no point at which the categories of inactive, active, and highly active are defined—this is a major omission as it is impossible to gauge how this compares to, for example, World Health Organization or other national guidelines in terms of minutes physical activity per week or metabolic equivalent minutes (apologies if this is indeed in the paper and I have missed it).

2. Demographics: although the authors should be commended for looking at differences between gender and age, there is no comment on socioeconomic status. For example, earlier in the paper, when describing the university, it would be useful to know what the demographics of the student population are (ie, do they represent general society or higher socioeconomic status?) This is important, as socioeconomic status (in the United Kingdom at least) is a major driver of physical activity. It would be useful for the reader as to how the subject population compares with the general population.

3. It is unclear to me how the metabolic equivalent minutes values of the subject population relate to that of the general population, and internationally. Over 4000 metabolic equivalent minutes per week is several times over World Health Organization guidance, and I would expect some analysis of how and why this might be the case.

4. In the discussion, there is a lot of description of the results from previous studies, and comparison with the current study, but without any analysis as to why there are similarities or differences. I also felt there was no real incorporation of the perceptions into the discussion, and no real analytical depth.

5. In the discussion, there is no real discussion of the limitations of the approach used, and no contextual framing of the findings.

#### Minor Comments

Abstract; objectives: Line beginning “the study also aims...” not quite clear: perhaps “This study also aims to capture student perceptions about the balance between curricular activities and leading a physically active lifestyle...”?

Introduction: (a) “being overweight” rather than overweight; (b) it would be useful to describe briefly what the few studies regarding students show.

Methods: validation of the new tool—more information on this would be useful: does the Cronbach alpha number represent test-retest reliability? In which case, how was validity measured?

Data collection and data entry: “written consent was obtained from each of the participants”

How were outliers excluded? How did the authors define “erratic entries”? Is this according to International Physical Activity Questionnaire cleaning criteria?

Views and opinions of the students: I would want more description of the items where there was discrepancy.

Table 8: there is a comment at the end of this section regarding why the authors feel students in different faculties are performing different levels of physical activity. This belongs in the discussion.

Discussion: the study on pooled data: was this from university students?

Tables: Table 4: why was a Mann Whitney Test used if the data presented are in mean SD (ie, if the data are nonparametric, shouldn’t the median IQR be used?)

Tables 6-8: it would be helpful to have the questions in the table to enable the reader to better see how they relate.
